# Inhibition of Biofilm Formation and Virulence Factors of Cariogenic Oral Pathogen Streptococcus mutans by Shikimic Acid

**DOI:** 10.1128/spectrum.01199-22

**Published:** 2022-07-26

**Authors:** Zhong Zhang, Yang Yang, Qun Sun, Weicai Zeng, Yuqing Li

**Affiliations:** a State Key Laboratory of Oral Diseases, National Clinical Research Center for Oral Diseases, West China Hospital of Stomatology, Sichuan University, Chengdu, China; b Department of Food Engineering, Sichuan University, Chengdu, China; c Key Laboratory of Bio-Resources and Eco-Environment of the Ministry of Education, College of Life Sciences, Sichuan University, Chengdu, China; Ohio State University

**Keywords:** natural compound, shikimic acid, anti-biofilm agent, glucosyltransferase, dental biofilm

## Abstract

Streptococcus mutans is known as an important oral pathogen causing dental caries, a widespread oral infectious disease. S. mutans synthesize exopolysaccharide (EPS) using glucosyltransferases (Gtfs), resulting in biofilm formation on the tooth surface. Bacterial cells in the biofilms become strongly resistant to a harsh environment, such as antibiotics and host defense mechanisms, making biofilm-based infections difficult to eliminate. Discovering novel antibiofilm agents, especially from natural products, helps to develop effective strategies against this kind of diseases. The present study investigated the inhibitory effect of shikimic acid (SA), one abundant compound derived from *Illicium verum* extract, on the biofilm formation of S. mutans. We found SA can reduce the EPS synthesized by this oral pathogen and modulate the transcription of biofilm formation related genes, leading to fewer bacterial cells in its biofilm. SA also interacted with cell membrane and membrane proteins, causing damage to bacterial cells. *Ex vivo* testing of biofilm formation on bovine teeth showed SA strongly decreased the number of S. mutans cells and the number of EPS accumulated on dental enamel surfaces. Moreover, SA exhibits almost no toxicity to human oral cells evaluated by *in vitro* biocompatibility assay. In conclusion, shikimic acid exhibits remarkable antibiofilm activity against S. mutans and has the potential to be further developed as a novel anticaries agent.

**IMPORTANCE** Natural products are an important and cost-effective source for screening antimicrobial agents. Here, we identified one compound, shikimic acid, from *Illicium verum* extract, exhibiting antimicrobial activity against S. mutans proliferation. It also inhibits biofilm formation of this bacteria through decreasing Gtf expression and EPS synthesis. Furthermore, this compound exhibits no significant cytotoxicity at its MIC against S. mutans, providing evidence for its clinical application.

## INTRODUCTION

Dental caries is a widespread oral infectious disease. The development of dental caries is closely associated with cariogenic biofilm formation of oral pathogens on tooth surfaces (1). Streptococcus mutans exhibits strong biofilm forming ability, which is considered an important cariogenic oral pathogen (2). This Gram-positive bacterium synthesizes glucans for adhesion and interacts with other bacteria species, resulting in the formation of a complex dental biofilm, thus promoting dental caries (3–5). Biofilm is a community of bacteria cells attached tightly to a surface and encapsulated in a self-secreted extracellular matrix, which can effectively protect bacterial cells from a harsh environment. Compared with planktonic cells, bacteria in biofilms are strongly resistant to antimicrobial agents and host immune defense mechanisms, making biofilm-associated infections hard to prevent and treat (6, 7). S. mutans utilizes several virulence factors to promote the formation of cariogenic biofilms and the development of dental caries. Glucosyltransferases (Gtfs) are important virulence factors of S. mutans. Encoded by three genes (*gtfB*, *gtfC*, and *gtfD*), these enzymes utilize sugars to synthesize glucans, thus promoting the binding of bacterial cells and the formation of a biofilm (8, 9).

Several methods have been developed to control dental caries. Regular toothbrushing and flossing are prevalent methods for caries prevention. However, these mechanical procedures could not eliminate oral pathogens including S. mutans (10). A proper strategy is to combine them with antimicrobial agents for regular dental hygiene, such as chlorhexidine, sodium fluoride, metal nanoparticles, and antimicrobial peptides, and these agents are capable of reducing the population of cariogenic bacteria in biofilms (11–13). However, these antimicrobial agents are not flawless. Consumption of chlorhexidine promotes the formation of dental calculus and the staining of teeth (14), while sodium fluoride and antibiotics cannot get rid of the development of bacterial resistance (15). Aggregation of metal nanoparticles in the oral environment leads to the loss of nanoscale activity, and some of the metal ions dissolved in saliva exhibit cytotoxicity for human oral cells (16). Moreover, biodegradation of antimicrobial peptides in saliva decreases its antimicrobial activity (12). It is clear that more effective agents for clinical caries treatment should be discovered.

With the extensive use of antimicrobial agents, more and more bacteria become resistant to certain antibiotics, and drug resistance is almost a century-long problem (17, 18). Discovering novel antimicrobial agents from natural compounds helps to provide effective strategies for clinical microbial prevention (19). *Illicium verum* (star anise) is widely used as a spice in cooking. It is also famous as the source of shikimic acid (3,4,5-trihydroxy-1-cyclohexene-1-carboxylic acid; SA), a starting molecule for the synthesis of oseltamivir (Tamiflu), an antivirus drug treating influenza (20, 21). SA exhibits several biological effects, such as antioxidant activity, anti-inflammatory activity, analgesic activity, anticoagulant activity, and antithrombotic activity (22, 23). However, only a few studies focus on the effect of SA on microorganisms. Bai et al. reported the antimicrobial activity of shikimic acid on staphylococcus aureus planktonic cells and biofilm formation by causing membrane damage (24–26). Considering the biologically multifunctional characteristic of SA, elucidating the effect of this natural compound on oral pathogens would provide evidence for the potential of its further clinical use.

This study aimed to investigate the antimicrobial activity of SA against S. mutans and the relevant mechanism of its action by evaluating its impact on biofilm formation, biofilm degradation, synthesis of extracellular polysaccharides (EPS), expression of virulence genes, and its direct interaction between bacterial surface structures. Biocompatibility of SA was also assessed by evaluating its cytotoxicity to human oral cells.

## RESULTS

### *Illicium verum* extract inhibits the proliferation and biofilm formation of S. mutans.

**FIG 1:**
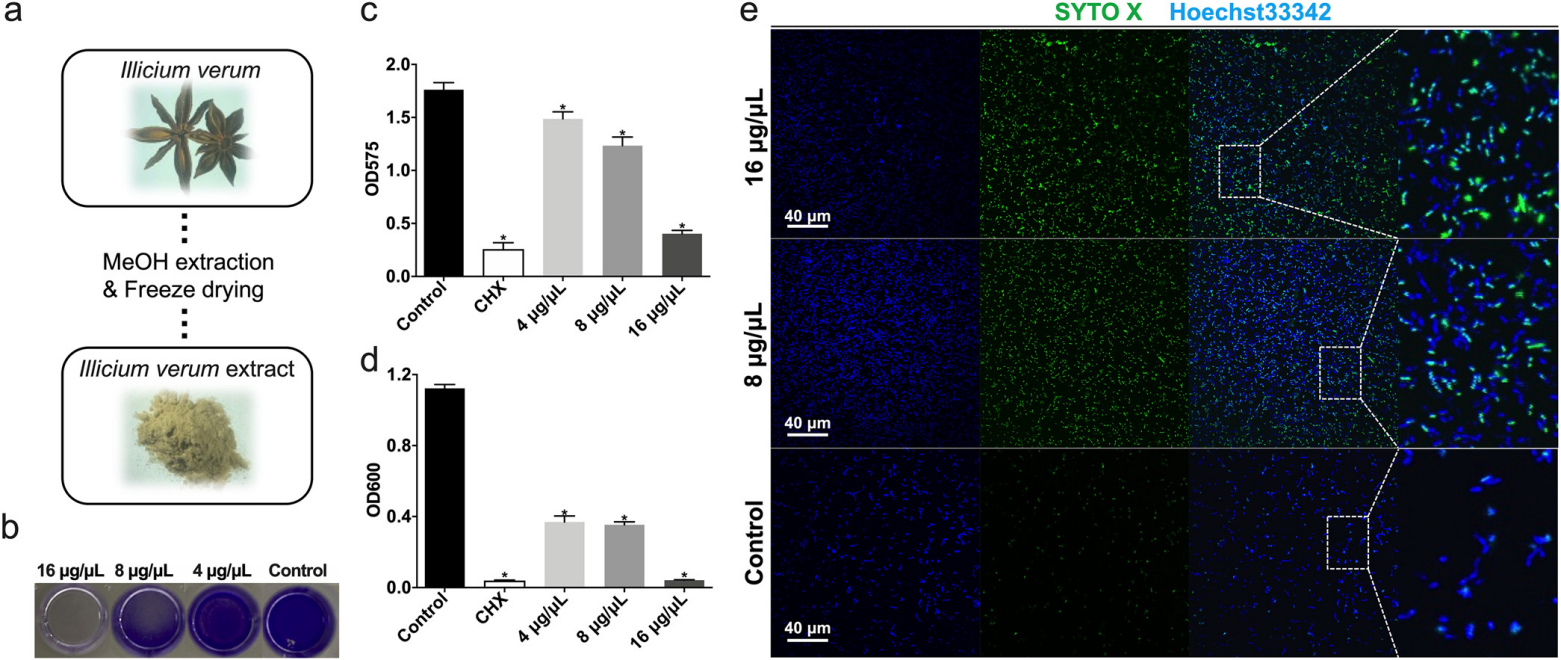
*Illicium verum* extract exhibits antimicrobial activity against *S. mutans*. (a) The brief description of *Illicium verum* extract preparation. (b–c) Effect of *Illicium verum* extract on S. mutans biofilm formation. Extract was added into bacterial suspension and cocultured for 24 h to assess biofilm formation. (c) Crystal violet staining of S. mutans biofilm treated with *Illicium verum* extract or chlorhexidine (CHX). (d) Effect of *Illicium verum* extract on planktonic S. mutans. Bacterial cells were cocultured with drugs for 24 h to assess antimicrobial activity. The concentration of 0.2% (wt/vol) chlorhexidine (CHX) was used as a positive control in the antimicrobial assays (c and d). (e) Live and dead staining of planktonic S. mutans cells treated with *Illicium verum* extract. SYTO X marks the dead cells, while Hoechst33342 marks all bacterial cells.

Various kinds of solvents could be utilized to extract the chemical components of plant materials. A common workflow is to dry the plant material followed by grinding and soaking in ethanol or methanol. Then, the extract was freeze-dried and collected. In this study, the *Illicium verum* extract was prepared by methanol extraction method followed by freeze drying (Fig. 1a). We then dissolved the extract powder in water and treated the bacterial cells in indicated concentrations. The bacteria were treated by SA for 24 h and then applied to analysis. According to the results of *in vitro* antimicrobial assays, *Illicium verum* extract showed antimicrobial activity at above 4 μg/μL. The MIC as well as minimal bactericidal concentration (MBC) of *Illicium verum* extract is about 16 μg/μL, as this extract almost inhibited the proliferation and biofilm forming of all S. mutans cells at this concentration (Fig. 1b to d). The result of confocal microscopy also showed the bactericidal effect of *Illicium verum* extract, as more SYTO X-positive cells can be seen in the images of 16 μg/μL and 8 μg/μL concentration groups compared with control group (Fig. 1e), indicating more dead bacterial cells captured. These dead cells tend to sink to the bottom of the bacterial culture and therefore be captured by microscopy. These data demonstrated the inhibitory effect of *Illicium verum* extract on S. mutans proliferation and biofilm formation.

### One abundant component of *Illicium verum* extract, shikimic acid (SA), inhibits the biofilm formation of S. mutans standard strain and clinical isolates.

**FIG 2:**
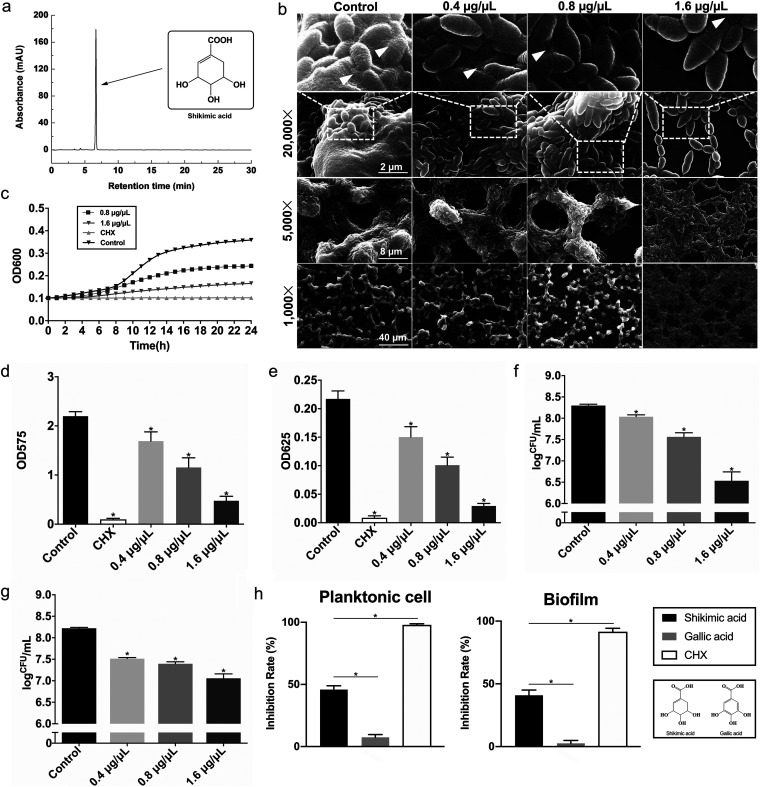
Shikimic acid inhibits *S. mutans* proliferation and biofilm formation. The concentration of 0.2% (wt/vol) chlorhexidine (CHX) was used as a positive control in the antimicrobial assays (c, d, e, and i). (a) HPLC-mass spectrometry identification of shikimic acid as the most abundant compound in *Illicium verum* extract. (b) SEM images of S. mutans UA159 24-h biofilms on glass coverslips. Images were taken at 1,000, 5,000, and 20,000× magnification, respectively. White triangles indicate the EPS produced by S. mutans. (c) A typical growth curve of S. mutans under the treatment of shikimic acid. The concentrations of 0.8 and 1.6 μg/μL of shikimic acid were incubated with S. mutans for 24 h. (d) Effect of shikimic acid on S. mutans biofilm formation assessed by crystal violet staining. (e) Quantitative measurement of water-insoluble EPS by anthrone assay. (f) Effect of shikimic acid on S. mutans biofilm formation assessed by measuring the average number of CFU in biofilms. (g) Effect of shikimic acid on S. mutans mature biofilms by the determination of the average number of CFU in biofilms. The S. mutans biofilms were cultured for 24 h without shikimic acid treatment, and the data were collected 24 h after the addition of shikimic acid. (h) Molecular structures of shikimic acid and one of its derivatives, gallic acid. (i) The antimicrobial activity of 0.8 μg/μL shikimic acid or gallic acid on S. mutans planktonic cells or biofilms. Values represent the means standard deviations from three independent experiments. *, *P < *0.05 compared with the untreated control.

The high-performance liquid chromatography (HPLC) followed by mass spectrometry (MS) is widely used to analyze the chemical components of certain mixtures, including plant extract. In this study, HPLC-MS and nuclear magnetic resonance (NMR) identified SA as the most abundant component in *Illicium verum* extract (Fig. 2a). The morphology of S. mutans biofilm on glass coverslips under the treatment of SA was measured by SEM. S. mutans occupied the surface of glass coverslips and formed dense, multilayered biofilms (Fig. 2b). The bacteria cells were covered in huge amounts of EPS as indicated. Compared with the control group, SA treated groups had less bacteria and EPS on glass coverslips. Treatment of the maximum concentration of SA in this assay (1.6 μg/μL) resulted in a thin layer of bacterial cells accumulated on glass coverslips with only a robust amount of EPS present in the border of cells (Fig. 2b). These images suggested that SA decreased S. mutans EPS synthesis and bacterial accumulation on glass coverslips. The antimicrobial activity of SA on S. mutans planktonic cells was revealed by determining the growth curve of S. mutans (Fig. 2c). SA decreased the total biomass of S. mutans biofilm, especially the 1.6 μg/μL group (Fig. 2d), revealed by crystal violet staining. SA also reduced both the amounts of EPS (Fig. 2e) and CFU counts (Fig. 2f) in S. mutans biofilm when introduced with bacterial dilution to form biofilms. As for the biofilm degradation assay, we added the agent in the culture media after the formation of bacterial biofilm, and the result showed SA decreased the number of live bacteria cells in S. mutans mature biofilm (Fig. 2g). To compare the antimicrobial activity between SA and its chemical derivatives, we used one of its structural analogues, gallic acid (Fig. 2h), for the antimicrobial assays on both planktonic cells and bacterial biofilms (Fig. 2i). It is worth noting that shikimic acid had a significantly stronger antimicrobial effect on S. mutans with nearly 50% of inhibition rate compared with gallic acid, indicating the dominating role of its chemical structure in its biological functions.

**FIG 3:**
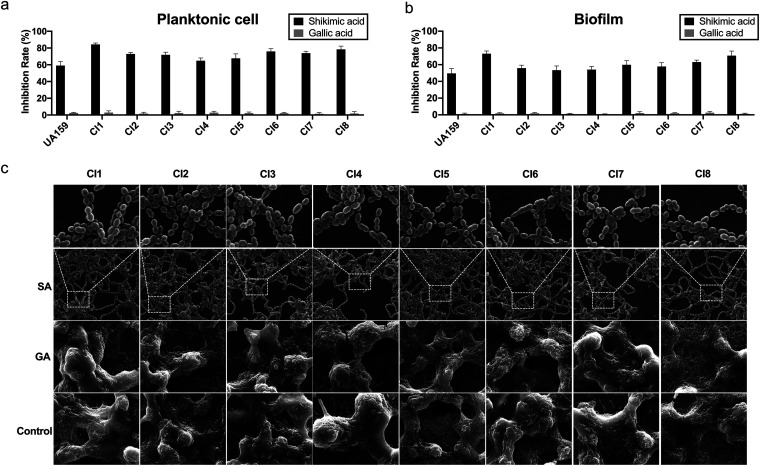
Antimicrobial activity of shikimic acid on S. mutans clinical isolates. (a) The inhibitory effect of 0.8 μg/μL shikimic acid or gallic acid on planktonic cell growth of S. mutans clinical isolates. (b) The inhibitory effect of 0.8 μg/μL shikimic acid or gallic acid on biofilm formation of S. mutans clinical isolates. (c) SEM images of the 24-h biofilms of S. mutans clinical isolates on glass coverslips. The bacteria were treated with 1.6 μg/μL shikimic acid or gallic acid. Values represent the means standard deviations from three independent experiments. *, *P < *0.05 compared with the untreated control.

To measure the antimicrobial activity of SA on other S. mutans strains, we further assessed its inhibitory effect on 8 S. mutans clinical isolates. The description of these strains is shown in Table 1. As a result, all 8 clinical isolates showed suppressed planktonic cell growth and biofilm formation when treated with 0.8 μg/μL of SA, while the same concentration of gallic acid showed little inhibitory effect on these strains (Fig. 3a and b). The SEM images of these clinical isolates showed a similar pattern as standard strain under the treatment of 1.6 μg/μL of SA, while 1.6 μg/μL of gallic acid had no significant inhibitory effect on the biofilm formation of S. mutans clinical isolates (Fig. 3c). In the control and gallic acid treated groups, S. mutans cells were covered in dense EPS and formed white pellet-like biofilm structures on coverslips. Upon SA treatment, S. mutans cells formed thin layered biofilms with little EPS produced (Fig. 3c). These results further verified the inhibitory effect of SA on S. mutans strains.

**TABLE 1:** Description of S. mutans clinical isolates used in this study

Strain	Preservation no.	Serological type	Collection place
CI1	3E034P	3e	West China Hospital of Stomatology
CI2	1F026P	1f	West China Hospital of Stomatology
CI3	2K018P	2k	West China Hospital of Stomatology
CI4	3E106S	3e	West China Hospital of Stomatology
CI5	3E009P	3e	West China Hospital of Stomatology
CI6	3E106P	3e	West China Hospital of Stomatology
CI7	2K030P	2k	West China Hospital of Stomatology
CI8	3E103S	3e	West China Hospital of Stomatology

### Quantitative measurements of bacteria and EPS in biofilms.

**FIG 4:**
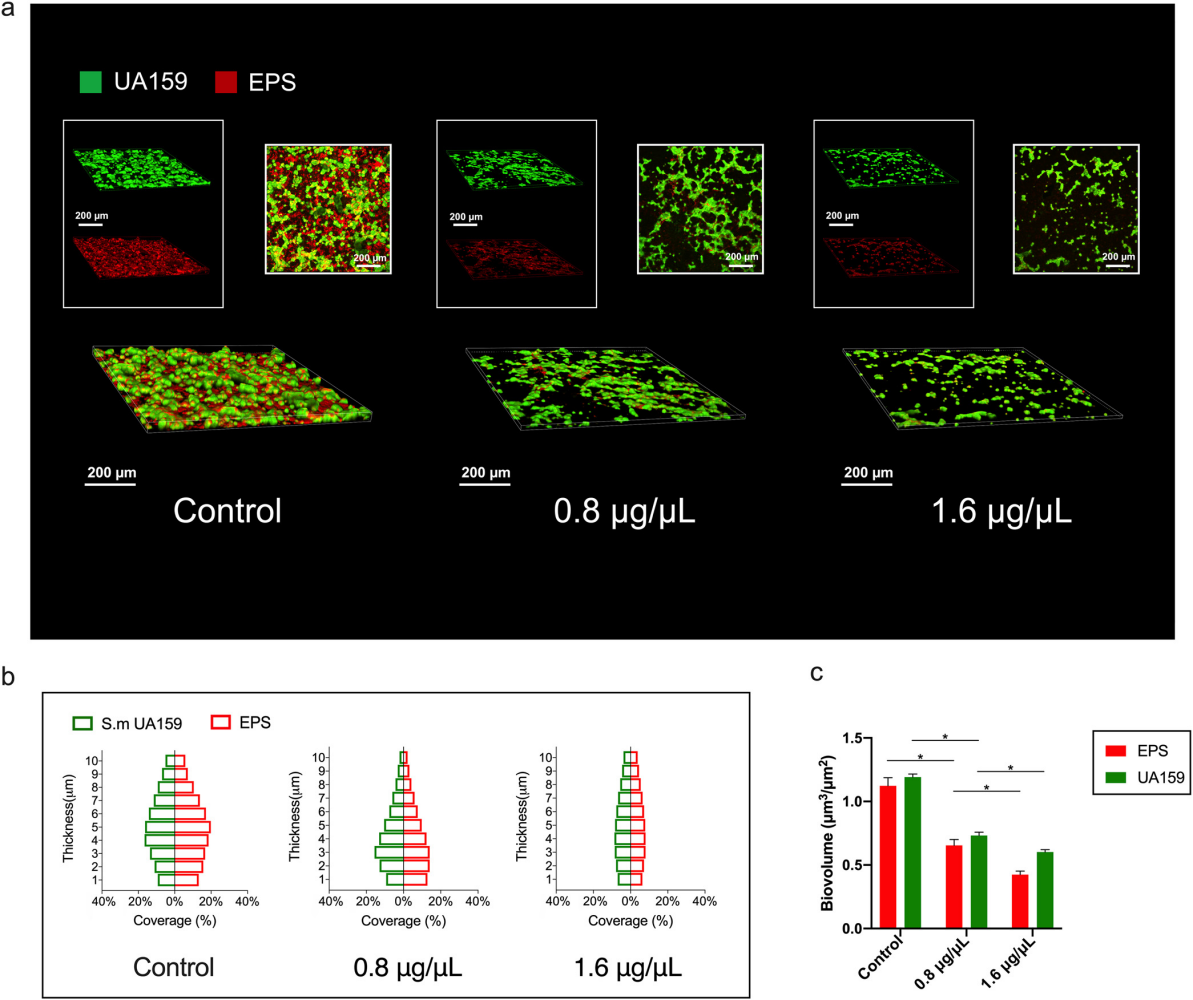
Shikimic acid decreases the amounts of bacteria and EPS in S. mutans biofilms. (a) Three-dimensional visualization and double-labeling imaging of S. mutans biofilm formed on glass coverslips. The green fluorescence (SYTO9) marks the live bacteria, while the red fluorescence (Dextran 647) marks the EPS synthesized by S. mutans. (b) The distribution of bacteria and EPS inside S. mutans biofilms. (c) Biovolume of bacteria and EPS. *, *P < *0.05 between compared groups.

Confocal laser scanning microscopy (CLSM) was used to evaluate the inhibitory effect of SA on EPS synthesis by S. mutans. The biofilms were cultured on glass coverslips for efficient image capture and data collection. SA were added immediately after bacterial dilutions were added into the culture plate, and the images were captured 24 h later. Fig. 4a shows three-dimensional images of S. mutans biofilms with green-labeled bacteria and red-labeled EPS. These micro-images revealed that treatment of SA reduced the amounts of both EPS and bacteria in S. mutans biofilms, which was in accordance with the result of SEM (Fig. 2b). Moreover, the distributions of bacteria and EPS from the bottom to the top of the S. mutans biofilms and the relevant biomass were quantified using Image J COMSTAT (Fig. 4b and c) according to reported method (27). The result statistically demonstrated the inhibitory effect of SA on S. mutans biofilm formation. These images and data suggested that either the proliferation of bacteria or the synthesis of EPS were inhibited in SA treated groups, resulting in less accumulation of biofilm on glass coverslips.

### SA deceases the expression level of Gtfs.

**FIG 5:**
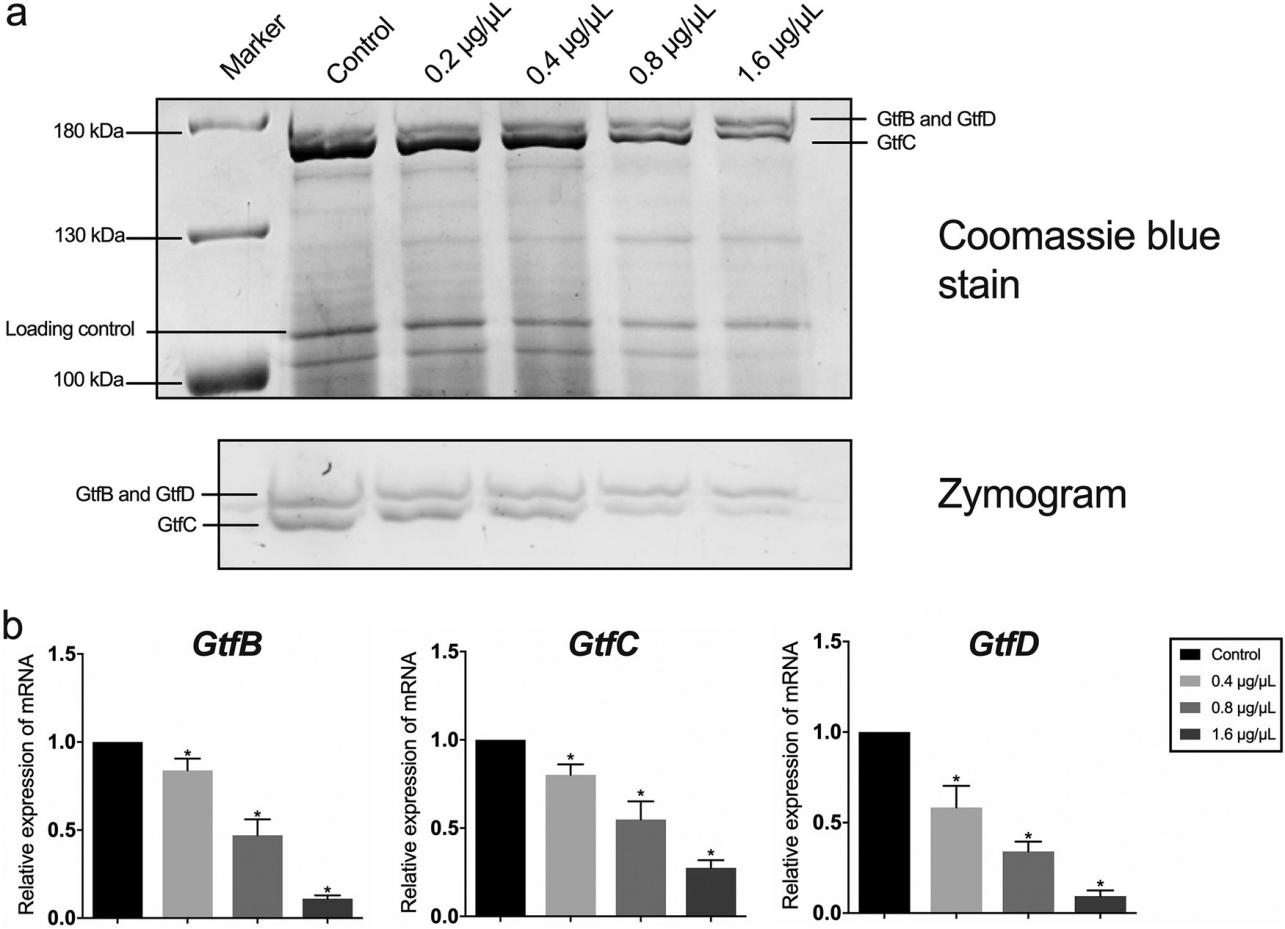
Shikimic acid decreases the transcription level of Gtf-encoding genes. (a) Enzymatic activity of Gtfs measured by zymogram assay. Gtfs were separated by SDS-PAGE. One gel was stained by Coomassie blue (upper gel), and the other gel was coincubated with sucrose and dextran T70 (lower gel). Glucan bands synthesized by GtfB, GtfD, and GtfC were marked in zymography, respectively. (b) Relative expression of *gtfB*, *gtfC*, and *gtfD* measured by qRT-PCR. S. mutans UA159 16S rRNA was used as the internal control. Values represent the means standard deviations from three independent experiments. *, *P < *0.05 compared with the control group.

The zymogram assay is widely used to detect the enzymatic activity of certain proteins, as the pattern of the products synthesized by certain enzymes and its substrates changed with the amount and activity of enzymes in gel. In this study, this assay was conducted to measure the amount of Gtf proteins. Dextran was added into the gel as the substrate of Gtfs. The gel was incubated at 37°C overnight followed by gel image analysis. The white bands inside the gels indicated the polysaccharides synthesized by Gtfs. The corresponding bands in gel (Fig. 5a, top) showed a typical pattern of Gtfs zymogram image in the control group, with two glucan bands close to each other. The upper band represents the glucans synthesized by GtfB (166 kDa) and GtfD (163 kDa), while the lower band represents the glucans synthesized by GtfC (153 kDa). The result demonstrated that the amount of Gtfs was decreased after the treatment with SA, especially in 1.6 μg/μL and 0.8 μg/μL groups (Fig. 5a). We then looked further into the expression of *gtf* genes in S. mutans treated with SA, and the result showed expression levels of *gtf* genes in the SA treated groups were downregulated compared with the control group (Fig. 5b), showing the inhibitory effect of SA on the expression of Gtfs.

### SA physically interacts with bacterial cells.

**FIG 6:**
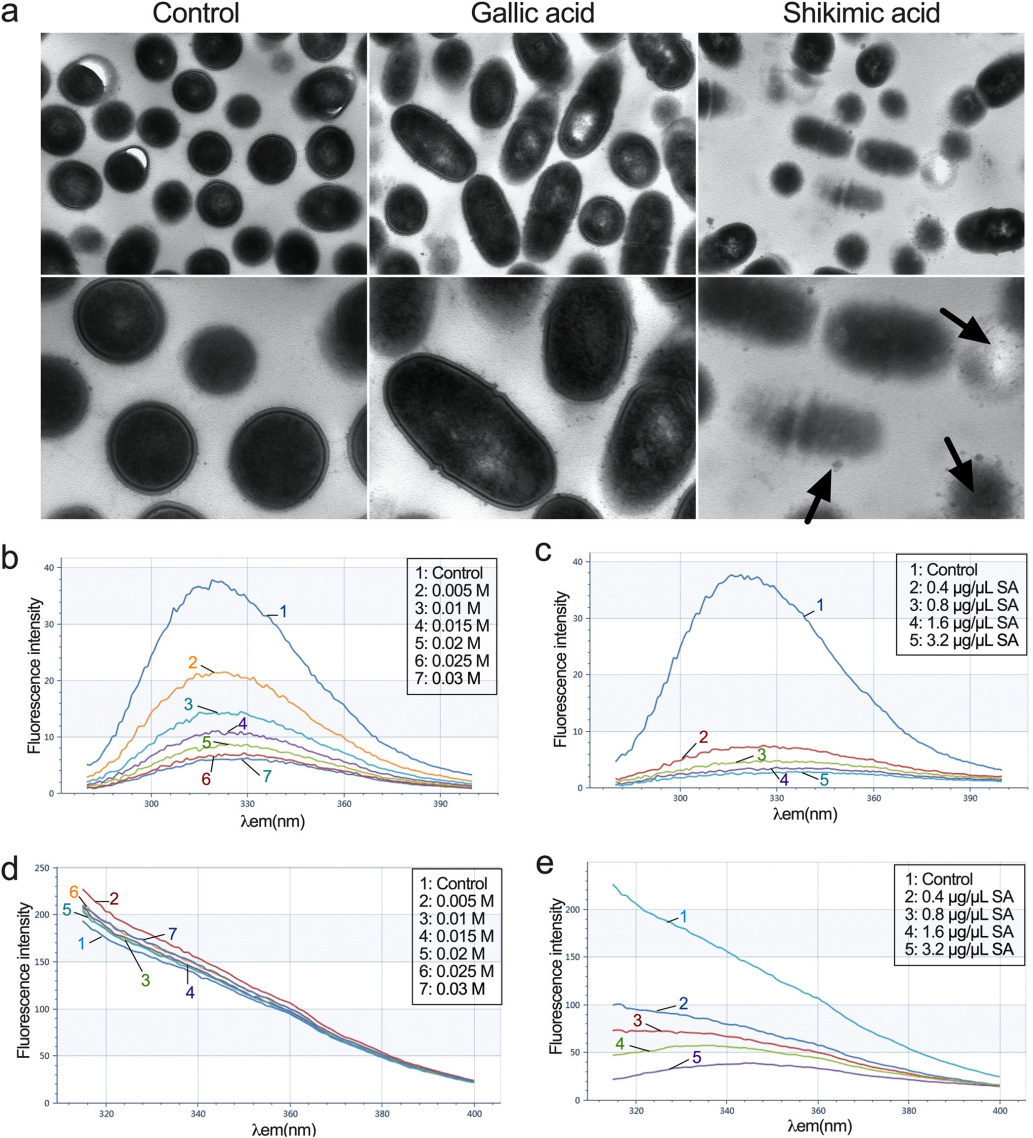
Shikimic acid interacts with the subcellular structures of S. mutans. (a) Transmission electron micrographs of S. mutans UA159. The bacteria were treated with 0.8 μg/μL shikimic acid or gallic acid. The black arrows point to the damage of membrane. (b) Effect of KI on fluorescence intensity of the membrane proteins of S. mutans at λex 258 nm. (c) Effect of shikimic acid on fluorescence intensity of the membrane proteins of S. mutans at λex 258 nm. (d) Effect of KI on fluorescence intensity of the membrane proteins of S. mutans at λex 296 nm. (e) Effect of shikimic acid on fluorescence intensity of the membrane proteins of S. mutans at λex 296 nm.

The transmission electron microscopy (TEM) images of S. mutans showed the membrane damage caused by shikimic acid. The membrane structures of S. mutans changed with the treatment of SA, compared with the control groups (Fig. 6a). To measure the interaction between SA and the bacterial membrane proteins, we conducted a fluorescence assay as reported by Bai et al. (24, 25). The existence of tryptophan (Trp), tyrosine (Tyr), and phenylalanine (Phe) residues on cell membrane contributes to its fluorescence characteristic. This characteristic can change when these residues are situated inside the membrane proteins or on the surface. Potassium iodide (KI) is widely used to examine the location of membrane proteins due to the direct binding effect of I- with amino acid residues. The direct interaction between SA and S. mutans surface proteins (or surface amino acid residues) could be revealed by the change of emission fluorescence spectra of S. mutans dilution in the presence of certain concentrations of SA. We first treated the S. mutans dilution with a series of concentrations of KI. The excitation wavelengths of Tyr and Phe residues were set at λex 296 nm and λex 258 nm as reported (24, 25). The results showed the emission fluorescence spectra of Phe residues are greatly affected by SA, suggesting that these residues are located mostly on the surface of membrane proteins (Fig. 6b). On the other hand, the emission fluorescence spectra of Tyr residues have only robust changes upon SA treatment, suggesting that these residues are mostly located inside of membrane proteins (Fig. 6d). We then treated the S. mutans dilution with SA. As the concentration of SA went up, the emission fluorescence intensity of Phe as well as Tyr dropped significantly to a level much lower than KI treatment, suggesting that SA might change the conformation of membrane proteins and expose the residues located inside of proteins, as well as interacting with them (Fig. 6c and e). These results revealed the direct interactions between SA and the membrane proteins of S. mutans.

### SA inhibits S. mutans biofilm maturation on dental enamel surface.

**FIG 7:**
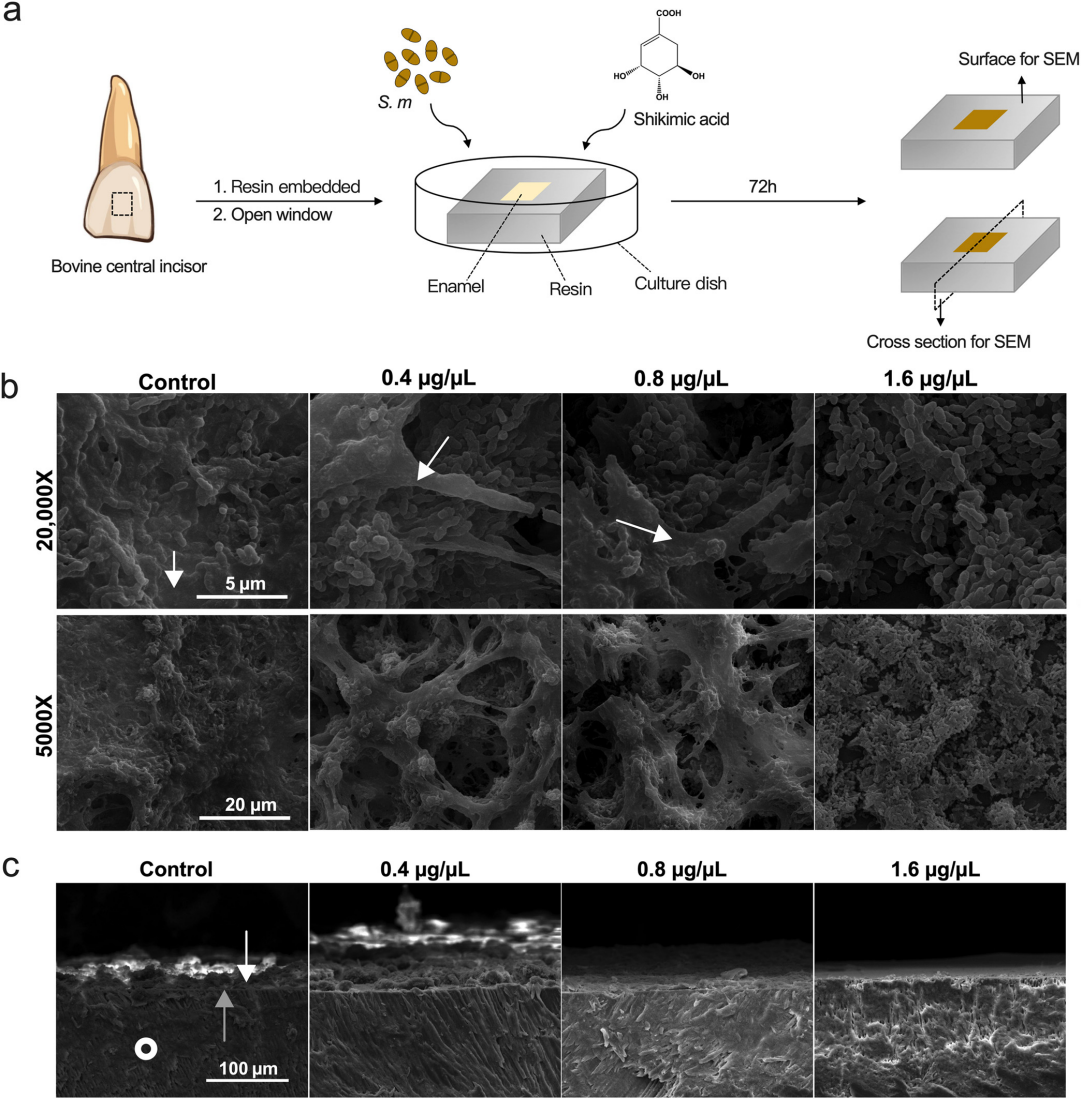
Shikimic acid inhibits S. mutans cells and EPS accumulation on dental enamel. (a) Brief description of *ex vivo* biofilm formation assay. (b) Surface images of S. mutans biofilm taken by SEM. EPS are indicated by the white arrows. (c) Cross-section images of S. mutans biofilm taken by SEM. S. mutans were treated with different concentrations of SA for 72 h and formed biofilms on bovine dental enamel surface. The white arrows point to the bacteria on the enamel surface, while the gray arrows point to the boundary between the cross section and the surface of enamel. White circles point to the morphology of enamel in the cross section.

The main procedures of this assay were illustrated in Fig. 7a in order to present a more understandable result. Since S. mutans is mainly colonized on the surface of tooth enamel, bovine central incisor enamel chips were utilized to assess the colonization and biofilm maturation of S. mutans to mimic the real conditions. We sectioned the bovine teeth into 5 mm by 5 mm by 1 mm chips and embedded them into resin, followed by trimming to open the enamel surface for bacterial colonization (Fig. 7a). The biofilms were cultured for 72 h in order to form matured, multilayered structures. SEM was used to investigate the S. mutans biofilms formed on the enamel surface, and both surface images as well as cross-section images were taken. Surface images showed that bacterial cells were in dense cover of EPS in control group, while the treatment of SA resulted in less EPS and fewer cells on the surface of bovine enamel (Fig. 7b). The view of the cross section of enamel showed that the 1.6 μg/μL of SA resulted in almost no structured biofilm formation on the enamel surface, and only a thin layer of biofilm was formed under the treatment of 0.8 μg/μL of SA (Fig. 7c), suggesting the inhibitory effect of SA on S. mutans EPS synthesis and biofilm formation.

### SA exhibits good biocompatibility to human oral cells.

In order to investigate the cytotoxicity of SA, we treated three cell lines with certain concentrations of SA for 24 h, similar to the treatment of S. mutans. The 1.6 μg/μL of SA exhibited good antimicrobial activity against S. mutans biofilm formation in our previous assays, but with no significant cytotoxicity against human oral keratinocytes (HOKs), RAW264.7 cells (RAWs) and periodontal ligament cells (PDLCs), which suggested that SA was biocompatible when applied to cultured cells to a certain extent.

## DISCUSSION

Dental biofilm is an important promoter of dental caries. Bacterial adhesion is of vital importance, and several strategies are applied by S. mutans to adhere to dental enamel (28, 29). In order to tightly adhere to the tooth surface, S. mutans utilize hydrophobic interactions between bacteria and dental hard tissue as well as synthesizing macromolecules to connect bacterial cells (30). Those macromolecules were glucans synthesized by GTFs, which are important virulence factors of S. mutans. Encoded by three genes known as *gtfB*, *gtfC*, and *gtfD*, GTF enzymes synthesize glucans by using sucrose (31). This process includes polymerization of sucrose glucosyl moieties and cross-linking of carbohydrates (28). In the present study, we found that SA could decrease the expression level of *gtf* genes, resulting in fewer GTF proteins and glucans synthesized by GTFs (Fig. 5). Except from SA, some other natural products also showed the inhibitory activity on the expression of *gtf* genes, including epigallocatechin gallate (EGCG), cinnamaldehyde, and naringenin, indicating a similar mechanism of antibiofilm effect of these agents (32–34).

In this research, the inhibition of EPS synthesis was also observed directly by microscopic methods. In our coverslip biofilm forming model, S. mutans cells in the control group were covered in dense EPS (Fig. 2b). In the SA treated groups, bacterial cells were not submerged in dense EPS, and bacterial cell boundaries were clearly visible. A similar phenomenon was also observed in our enamel biofilm forming model (Fig. 7b and c). CLSM result revealed that the amount of both live bacteria and EPS was decreased when treated with SA (Fig. 4). However, EPS tended to be suppressed more significantly (Fig. 4c). Those images demonstrated that less EPS was synthesized by S. mutans under the treatment of SA.

SA could damage the cell membrane of S. mutans, leading to the leakage of cell contents revealed by TEM (Fig. 6a). Bai et al. reported SA could damage the cell membrane of Staphylococcus aureus by using TEM, which is in accordance with our result (25). Moreover, we found SA can also interact with the membrane proteins of S. mutans. The fluorescence intensity of S. mutans treated with KI with the excitation wavelengths at 258 nm and 296 nm revealed that the Tyr residues were situated on the surface of membrane proteins, while the Phe residues were not (Fig. 6b and d). Moreover, SA could interact with the membrane proteins, causing the conformation change of these proteins and the decrease of fluorescence intensity (Fig. 6c). These results were in accordance those of Bai et al. (25). However, we found SA could decrease the fluorescence intensity at an excitation wavelength of λex 296 nm, indicating the situation change of Phe residues in membrane proteins caused by SA (Fig. 6e). This may due to the damaging effect of SA on S. mutans cells and the conformation change of membrane proteins when treated with SA.

SA decreased the expression level of three *gtf* genes related with biofilm formation as measured by qRT-PCR (Fig. 5b). This resulted in less EPS produced extracellularly, causing less biofilm formed. The results of CLSM and SEM also demonstrated this information (Fig. 2b and Fig. 4).

In this experiment, we grew S. mutans biofilms on bovine enamel chips (generated from bovine central incisors) in order to mimic the *in vivo* conditions. As a cariogenic bacterium, S. mutans colonize the enamel surface of teeth and promote caries development. Therefore, the enamel material could better mimic real conditions compared with glass coverslips, despite creating difficulties in data collection.

**FIG 8:**
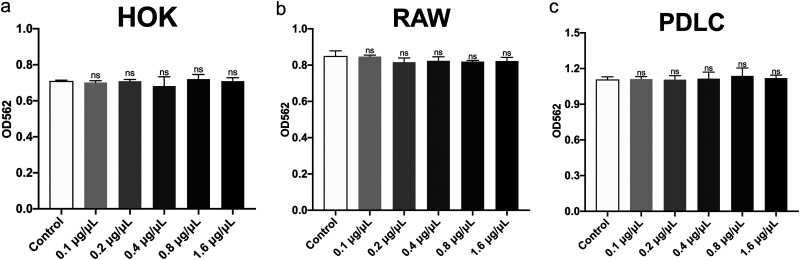
Biocompatibility of shikimic acid with several types of cells evaluated by CCK-8 assay. (a) Cytotoxicity of SA on human oral keratinocytes (HOKs). (b) Cytotoxicity of SA on RAW264.7. (c) Cytotoxicity of SA on human periodontal ligament cells (PDLCs). Values represent the means standard deviations from three independent experiments. *, *P < *0.05 compared with the untreated control. ns, not statistically significant compared with the untreated control.

When assessing a novel agent for application, biocompatibility is an important aspect that needs to be evaluated. In this study, the cytotoxicity of SA to HOKs, RAWs and PDLCs was measured to evaluate its biocompatibility for potential utilization. The result showed that treatment of SA leads to no significant difference in the proliferation of all the applied cells (Fig. 8), which demonstrated that SA exhibits good biocompatibility and is acceptable for application as a novel anticaries agent. However, the potential bioaccumulation of SA in the oral cavity may cause higher local concentration of this agent, which needs further consideration.

In the present research, we investigated and interpreted the antibiofilm activity of SA on the mRNA level of S. mutans. However, we didn’t find the specific molecular target interacting directly with SA in S. mutans cells and the relevant docking site. Understanding the detailed mechanism of this molecular interaction is challenging work, and we hope to solve it in future research. Moreover, many of the SA derivatives are commercially accessible and they might exhibit a similar effect as SA on oral pathogens. Future research can start with these molecules to study their effects on oral pathogenic microorganisms. As a natural antioxidant, SA showed effective antimicrobial activity against S. mutans and remarkable biocompatibility in this study. In an era of increasing resistance to antibiotics and a growing number of multidrug-resistant bacteria strains, natural compounds are apparently a great source of discovery of novel antimicrobial agents. Here, we want to draw attention to the discovery of novel antimicrobial natural compounds and their clinical applications.

In conclusion, this study identified one abundant compound from *Illicium verum*, SA, and investigated its antibiofilm activity against S. mutans, as well as the relevant mechanism of its action and its biocompatibility. Our results provide the evidence for this natural organic molecule as an anticaries agent.

## MATERIALS AND METHODS

### Chemicals, test bacteria, and growth conditions.

Shikimic acid and gallic acid were commercially accessed from Sigma, Inc. S. mutans UA159 were obtained from the American Type Culture Collection (ATCC). The bacteria were cultured overnight in Brain Heart Infusion (BHI) broth (BD, Sparks, MD, USA) at 37°C for further experiments. BHIS (BHI broth containing 1% [wt/vol] sucrose) was used for biofilm assays. The negative control group was set as sterile phosphate-buffered solution (PBS) of the same volume as SA. The concentration of 0.2% (wt/vol) chlorhexidine was used as a positive control. The inhibition rates of planktonic cells or biofilm refer to the difference between the absorbance of the experimental group and the negative control group as a percentage of the absorbance of the negative control group.

### Chemical identification of plant extract.

Extract was separated by preparative high-performance liquid chromatography (HPLC; Agilent 1290 Series Purification System; Agilent Technologies Inc., CA, USA) using a HPLC column (XB-NH_2_; Ultimate, Shanghai, China). The mobile phase contained acetonitrile (A) and deionized water with 1% phosphoric acid (B). The program of gradient elution consisted of 0–30 min, 90% A and 10% B. The analytical conditions were as follows: column temperature, 30°C; flow rate, 1 mL/min; injection volume, 2 μL (the concentration of the extract was 2 mg/mL); and detection, 210 nm (the reference wavelength: 360 nm). The primary compounds isolated from HPLC were characterized by HPLC-MS and NMR. The analytical conditions of HPLC-MS were identical to those of the HPLC analysis, and the mass spectra were performed using a Thermo Scientific TSQ Quantum Ultra (Thermo Fisher Scientific Inc., MA, USA) with an electrospray source operating in negative ion mode and the mass spectrum scanning range of 50–800 *m/z*. The ^1^H and ^13^C NMR spectra were recorded with a Bruker aV II 600 MHz spectrometer (Bruker Co., Ltd., Fällanden, Switzerland), and deuterium oxide (D_2_O) was used as the solvent.

### Crystal violet staining.

Crystal violet staining assay was performed to evaluate the effect of SA on biofilm formation of S. mutans according to a reported protocol with modifications (35). Briefly, an overnight culture of S. mutans was diluted using BHIS into a 24-well plate. SA was then added to each well, and the final concentrations of SA were 1.6 μg/μL, 0.8 μg/μL, and 0.4 μg/μL. The plate was incubated at 37°C anaerobically for 24 h. Then, excess medium was removed and the plate was washed twice using sterile PBS to remove loosely adherent cells. Biofilms in the wells were fixed using 4% (wt/vol) paraformaldehyde for 15 min. We then added 0.1% (wt/vol) crystal violet-glacial acetic acid solution to each well to stain for 5 min. After removing the solution, the plate was washed twice with sterile water. A 33% (vol/vol) acetic acid solution was added to dissolve the dye. Finally, 0.5 mL solution in each well was transferred to a new 24-well plate to read the absorbance at 575 nm of each well by a spectrometer (Powerwave XS2, Bio-Tek, USA). Crystal violet staining was performed in triplicate, and each concentration was tested in three wells in a single test.

### CFU counting.

The effect of SA on S. mutans biofilm formation and mature biofilm was evaluated by CFU counting as reported (27). For the biofilm formation assay, BHIS-diluted overnight cultures of S. mutans and SA were added into a 24-well plate. Final concentrations of SA were 1.6 μg/μL, 0.8 μg/μL, and 0.4 μg/μL. The plate was incubated anaerobically at 37°C for 24 h. Then, the excess medium was removed and the plate was washed twice with sterile water. The biofilms were scraped out by pipettes, resuspended in PBS, and plated onto BHI agar plates after a serial dilution from 10^4^-fold to 10^6^-fold. The plates were incubated anaerobically at 37°C for 2 days to determine the number of CFU.

For the mature biofilm assay, overnight cultures of UA159 diluted in BHIS were first added in a 24-well plate and were incubated anaerobically at 37°C for 24 h to form mature biofilms. SA was then added to each well, and the final concentrations of SA were 1.6 μg/μL, 0.8 μg/μL, and 0.4 μg/μL. The plate was incubated anaerobically at 37°C for another 24 h. Then, the medium was removed and the plate was washed 3 times with sterile PBS. The determination of CFU followed the same procedures as biofilm formation assay.

### Quantitative determination of water-insoluble EPS.

The inhibitory effect of SA on water-insoluble EPS synthesis of S. mutans was confirmed by anthrone-sulfuric method according to a reported protocol (36). Briefly, an overnight culture of S. mutans was diluted in a 24-well plate at 37°C in BHIS with SA. The final concentrations of SA were set as 1.6 μg/μL, 0.8 μg/μL, and 0.4 μg/μL. The plate was anaerobically incubated at 37°C for 24 h. The culture medium was carefully removed, and the biofilm was washed twice with sterile PBS. S. mutans cells in each well were resuspended in 1 mL of sterile PBS by vigorous pipetting, and the suspension was transferred to a sterile 1.5-mL centrifuge tube (Corning). The suspension was centrifuged at 6,000 *g*, 4°C for 10 min. The supernatant was then discarded, and the pellet was resuspended and washed 3 times with sterile PBS to remove all the water-soluble EPS. The water-insoluble EPS was extracted using 1.0 M NaOH with constant agitation for 2 h at 37°C. After then, the supernatant was mixed with 3 volumes of anthrone-sulfuric acid reagent and heated in a water bath at 99°C for 8 min until the reaction was completed. The concentration of carbohydrate was determined in the solution. The solution was then allowed to cool to room temperature, and its absorbance at 625 nm was measured in a 96-well plate using a microplate reader (BioTek).

### Biofilm formation assay on glass coverslips.

The surface morphology of S. mutans biofilm on glass coverslips was observed by SEM. After UV disinfection for 2 h, round-shaped glass coverslips were placed in a 12-well plate with S. mutans UA159 diluted suspension (10^5^ CFU/mL) and SA solution in each well. The experimental groups were set as above. The plate was anaerobically incubated at 37°C for 24 h. After the incubation, 2.5% glutaraldehyde (vol/vol) was added into the plate to fix the biofilms. Then, gradient concentrations of ethanol (30%, 40%, 50%, 60%, 70%, 80%, 90%, and 100%) were added for 15 min at each concentration in order to dehydrate the biofilms. The observation process was conducted by a scanning electron microscope (Inspect F, FEI, Netherlands). Three points were randomly selected in each glass coverslip.

### Confocal laser scanning microscopy (CLSM).

The bacteria and EPS in S. mutans biofilm were observed and quantified by CLSM. First, S. mutans diluted in BHIS was added into a 12-well plate with a glass cover slip in each well. Dextran 647 conjugate (Invitrogen, USA) and gradient concentrations of extract were then added. The final concentrations of SA were 1.6 μg/μL and 0.8 μg/μL. A bacterial dilution with PBS was used as the negative control. After anaerobic incubation for 24 h, the excess medium was removed and SYTO9 (Invitrogen, USA) was added for bacteria labeling. The observation process was conducted by a confocal laser scanning microscope (N-SIM, Nikon, Japan), and dual-channel scanning observation was applied. The micro-images were analyzed, and the amounts of bacteria as well as EPS were quantified using Image J COMSTAT software (NIH, USA) according to reported methods (27).

### Transmission electron microscopy (TEM).

The S. mutans cells were treated with 0.8 μg/μL of SA or gallic acid at 37°C for 12 h. The bacterial pellets were then treated as previously reported (37). The thin sections were observed by TEM (JEM-1400PLUS, JEOL, Tokyo, Japan).

### Membrane protein assay.

The effect of SA on membrane protein of S. mutans was determined as reported with modifications (24, 25). An overnight culture of S. mutans cells was resuspended in PBS to make the concentration of 10^9^ CFU/mL. We mixed 0.2 mL of bacterial suspension with 1.8 mL of KI or SA solution of different concentrations. After incubation for 1 h at room temperature, the fluorescence spectra were measured using a fluorescence spectrophotometer (SpectraMax iD5, Molecular Devices, CA, USA). The excitation wavelength was set at λex 258 nm or λex 296 nm, and the emission spectra were scanned from 280 to 400 nm.

### Quantitative real-time PCR (qRT-PCR).

S. mutans suspension was diluted in a 12-well cell culture plate using BHIS. SA was then added in each well. The final concentrations of SA were 1.6 μg/μL, 0.8 μg/μL, and 0.4 μg/μL. The plate was anaerobically incubated for 24 h, and the bacterial cells were resuspended by PBS and centrifuged at 4°C and 6,000 rpm. The total RNA of S. mutans UA159 was extracted using TRIzol reagent (Invitrogen, USA). The PrimeScript RT reagent kit (TaKaRa, Japan) was applied for reverse transcription. Relative expression levels of bacterial genes were measured using qRT-PCR. S. mutans UA159 16S rRNA was set as inner control. Primers were synthesized by TsingKe biotechnology, Inc. (The primer sequences are shown in Table 2.) PCR procedures were set as follows: denaturation at 95°C for 10 min; amplification for 45 cycles consisting of denaturation at 95°C for 10 s, annealing at 61°C for 32 s, and extension at 73°C for 32 s. qRT-PCR was performed using LC480 (Roche, USA). Relative mRNA expression levels of the genes were determined using the 2^−ΔΔ^*^CT^* method. qRT-PCR was performed in triplicate.

**TABLE 2:** Sequences of primers used for qRT-PCR in this study

Primers	Sequences
16S rRNA	Forward: 5′-CCATGTGTAGCGGTGAAATGC-3′
	Reverse: 5′-TCATCGTTTACGGCGTGGAC-3′
*gtfB*	Forward: 5′-AGCCGAAAGTTGGTATCGTCC-3′
	Reverse: 5′-TGACGCTGTGTTTCTTGGCTC-3′
*gtfC*	Forward: 5′-TTCCGTCCCTTATTGATGACATG-3′
	Reverse: 5′-AATTGAAGCGGACTGGTTGCT-3′
*gtfD*	Forward: 5′-TTGACGGTGTTCGTGTTGAT-3′
	Reverse: 5′-AAAGCGATAGGCGCAGTTTA-3′

### Zymogram assay.

The effect of SA on the activity of S. mutans glucosyltransferases (GtfB, GtfC, and GtfD) was determined by the zymogram assay as previously described (38) with modifications. In brief, diluted S. mutans suspension treated with SA solutions (final concentrations: 1.6 μg/μL, 0.8 μg/μL, 0.4 μg/μL, and 0.2 μg/μL) were grown under anaerobic conditions in 50 mL centrifuge tubes at 37°C for 8 h. After incubation, the tubes were centrifuged at 4°C and 13,000 rpm for 10 min to remove the bacterial cells. The supernatants were mixed 3:1 with ethanol and then centrifuged at 25,000 rpm for 20 min to collect the precipitates. An amount of 0.2 mL sterile PBS was added to each tube to resuspend the precipitates. These solutions were mixed 1:1 with loading buffer and loaded into two sodium dodecyl sulfate (SDS)-6% polyacrylamide gels for SDS-polyacrylamide gel electrophoreses (SDS-PAGE). After the SDS-PAGE process, Coomassie blue R-250 (Sigma) staining was applied to one gel for total protein detection, while the other was used for the zymogram assay to evaluate the enzymatic activity of Gtfs. To perform the zymogram assay, the gel was immersed in renaturing buffer containing 2.5% (vol/vol) Triton X-100 (Sigma) at 0°C. Then, it was anaerobically incubated in a mixed solution with 0.2 M Na_2_HPO_3_, dextran T70 (Sigma), and sucrose for 24 h. After incubation, the gel was washed twice using PBS, and the glucan bands that were synthesized by the Gtfs could be seen in the gel. A gel image analyzing system (Chemidoc MP, Bio-Rad, USA) was used to capture the images.

### Biofilm formation assay on bovine dental enamel surface.

In order to be more in accordance with the actual situation, an *ex vivo* assay based on bovine dental enamel biofilm formation was performed. Briefly, bovine central incisors were collected from local markets. The teeth were then washed with 75% (vol/vol) ethanol and sliced into square pieces with a length of 4 mm. These enamel pieces were then embedded in epoxy resin. They were windowed and polished to expose the enamel surface for bacterial adhesion. After UV disinfection for 2 h, these specimens were placed in a 12-well culture plate with diluted suspensions (10^5^ CFU/mL) of S. mutans. SA was added to each well to make final concentrations to 1.6 μg/μL, 0.8 μg/μL, and 0.4 μg/μL, respectively. A dilution with sterile PBS served as control. After incubation at 37°C for 72 h, the specimens were fixed with 2.5% (vol/vol) glutaraldehyde at 4°C for 12 h. Then, series concentrations of ethanol (30%, 40%, 50%, 60%, 70%, 80%, 90%, and 100%, vol/vol) were added into the plate for 15 min to dehydrate the biofilms. After gold spray, the specimens were observed using a scanning electron microscope (Inspect F, FEI, Netherlands). Three points were randomly selected for observation of each specimen.

### Biocompatibility.

The cytotoxicity of SA on human oral keratinocytes (HOKs), macrophages (RAW), and human periodontal ligament cells (HPDLCs) was evaluated by CCK-8 assay following the manufacturer’s instructions (Cell-Counting-Kit 8, Dojindo, Japan). HOKs cell line and RAW cell line were provided by the State Key Laboratory of Oral Diseases, Sichuan University, China. HPDLCs were isolated as previous reported (39). The periodontal ligament tissues of extracted third molars (all donors gave their informed consent, which was approved by the Ethics Committee of the Hospital of Stomatology, Sichuan University, China) were harvested, minced, and transferred to T25 flasks. The primary HPDLCs came from periodontal ligament tissues. The cells were cultured in α-MEM (Gibco, UK), 100 U/mL penicillin, and 100 mg/mL streptomycin supplemented with 20% fetal bovine serum (FBS; Gibco, UK) and cultured at 37°C under 5% CO_2_. After three passages, the cells were cultured in 96-well plates at a density of 5,000 cells/well and SA was added into the plates, and final concentrations of SA were 1.6 μg/μL, 0.8 μg/μL, 0.4 μg/μL, 0.2 μg/μL, and 0.1 μg/μL. The control group was set as above. After incubated at 37°C under 5% CO_2_ for 24 h, the cells were washed twice with sterile PBS, and 0.1 mL CCK-8 reagent was added into each well. The plates were then incubated for 3 h at 37°C. After incubation, the absorbance at 562 nm of each well was measured using a microplate reader (Powerwave XS2, Bio-Tek, USA). CCK-8 assays were performed in triplicate, and each concentration was measured in three wells in a single experiment.

### Statistical analyses.

Statistical analyses of the data were performed with SPSS software (IBM SPSS Statistics 25.0 for Mac OS; SPSS Inc., USA) using analysis of variance (ANOVA), followed by a *post hoc* Tukey’s test. Results were expressed as mean with SD. A *P* value < 0.05 was considered statistically significant.
